# Cross-Sector Policies for Achieving Equitable Obesity Prevention and Food Security: A Review of Real-World Actions

**DOI:** 10.1007/s13679-026-00738-z

**Published:** 2026-07-10

**Authors:** Christina Zorbas, Sally Schultz, Shu Wen Ng, Erica Reeve, Ayuba Issaka, Chioma Anidi, Kathryn Backholer

**Affiliations:** 1 Institute for Health Transformation, Global Centre for Preventive Health and Nutrition, School of Health and Social Development, Faculty of Health, Deakin University, 1 Gheringhap St, 3220 Geelong, Australia; 2 Gillings School of Global Public Health, University of North Carolina, Chapel Hill, USA

**Keywords:** Obesity prevention, Food security, Health equity, Social determinants of health, Food policy, Public policy

## Abstract

**Purpose of Review:**

This narrative review examines how government sectors beyond health contribute to the dual goals of equitable obesity prevention and food security through structural, cross‑sector policies. It aimed to synthesise real‑world evidence on the design, implementation and diet‑related impacts of policies across social, education, economic, trade and agricultural, and legal domains.

**Recent Findings:**

Emerging insights highlight the need for further evaluation of how income supports contribute to diet-related health; the expansion of school meal programs given their multiple co-benefits; the feasibility and impacts of adopting health-promoting taxes that generate public revenue for equity-focused initiatives; how agricultural supports can be repurposed to improve local and equitable food access; and the use of legal instruments to hold food industries accountable and advance public health interests.

**Summary:**

Coordinated, equitable progress on obesity prevention and food security will require enhanced policy coherence, effective accountability mechanisms, better data on diet and equity outcomes for cross-sector policies, and political commitment to overcome siloed and often unbalanced decision-making processes.

## Introduction

Dietary risks refer to patterns of foods and beverage consumption that can increase the risk of overweight, obesity and related non-communicable diseases (NCDs), which are among the leading global causes of illness and premature mortality [1]. These include insufficient intakes of nutrient-rich foods and beverages (e.g. wholegrains, fruit, legumes, vegetables, milk, seafood) and excessive intakes of energy-dense, nutrient-poor foods and beverages (e.g. red and processed meats, sugar-sweetened beverages (SSBs)) [1]. Inequalities in dietary risks and related health outcomes, such as overweight and obesity, persist across populations, with people in lower socioeconomic positions, those living in rural areas, First Nations and ethnic minority communities, among others, often disproportionately affected [2].

Dietary inequalities are influenced by interactions between people’s daily living environments (e.g. food retail, food marketing), social conditions (e.g. income, housing), and socio-political contexts (e.g. institutional laws and practices) that reinforce systemic inequity (i.e. classism, racism, under-representation) [3]. For instance, dietary intakes and access to healthy versus unhealthy food retailers vary by socioeconomic position and geography in high‑income countries such as Australia, and are further shaped by food insecurity, high food prices and lower incomes [4–8]. Historically examined separately, the relationship between food insecurity–the lack of sustained access to sufficient nutritious food–and overweight and obesity has become increasingly evident in the literature. A meta-analysis of studies published up until 2021 (*n* = 36,113 adults and children) found that people experiencing food insecurity had 1.5 times the odds of experiencing obesity compared to food secure people [9]. Qualitative evidence indicates that people experiencing food insecurity often rely on energy-dense, nutrient-poor options due to their relative affordability and accessibility [10].

**Fig. 1:**
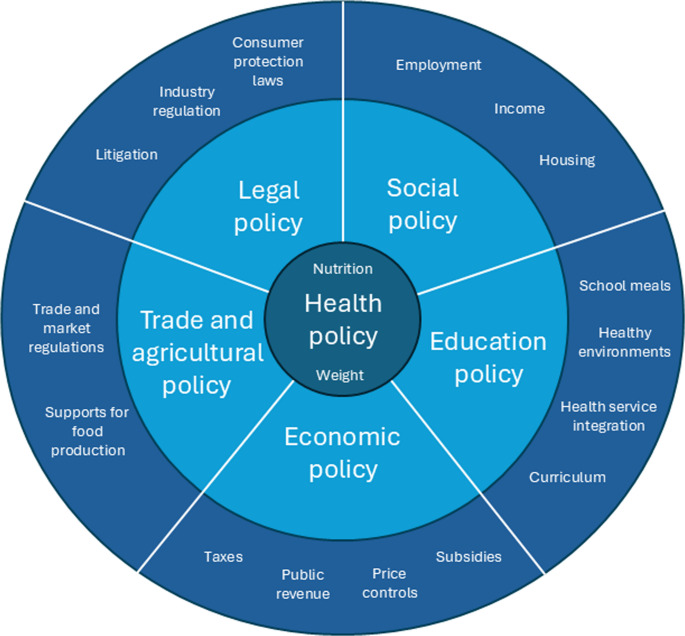
Illustrative cross-sector policy domains that can support the dual goals of equitable obesity prevention and food security

Given the common structural drivers of food insecurity and overweight and obesity, governments have multiple opportunities to implement population-level policies that create healthier environments, conditions and contexts, moving away from dominant narratives that frame food intake and obesity primarily as matters of individual choice [11]). Fundamentally, this will necessitate policies beyond the health sector. In 2021, the World Health Assembly adopted a resolution to reduce the burden on NCDs, urging Member States to *“apply whole-of-government and whole-of-society approaches that place achievement of … obesity-related global voluntary targets at the centre of the response.”* [12] Multiple frameworks highlight the importance of designing and implementing policies across sectors to improve population diets [13–15]. Figure 1 illustrates cross-sector policy domains identified across these existing frameworks, including strengthening social protection (such as income supports, housing and employment), improving access to healthy foods in educational settings; reshaping economic structures such as taxation and public investment; repurposing current agriculture supports towards healthy and sustainable foods systems practices; and strengthening legal and regulatory systems to protect consumers from potentially harmful commercial practices [13–15].

Current frameworks also include other sectors, such as transport and planning, environment, business and communications in conceptualising action on obesity prevention and food security. However, gaps remain in moving beyond conceptual models to synthesising evidence on real-world cross-sectoral policies across critical policy domains.

### Review Aim and Methods

We aimed to synthesise the literature on cross-sectoral policies that are shaped by government sectors beyond health, in terms of their design, implementation and diet-related health impacts among priority communities worldwide, where evidence was available. A targeted narrative review approach was adopted, whereby authors identified and synthesised contemporary real-world policy examples across the key domains in Fig. 1. Evidence was drawn from peer‑reviewed studies, policy reports, grey literature, and national case studies. Policy examples were selected based on their relevance to the review objectives, the availability of information regarding policy design and implementation, evidence of impacts on diet-related health outcomes or their determinants, and a deliberate effort to include examples implemented across both high-income and low- and middle-income countries. Diet‑related outcomes included any indicators of diet, nutrition, weight or Body Mass Index (BMI), food insecurity, and broader societal impacts such as healthcare costs. This targeted narrative review approach enabled integration of diverse study designs and data sources, which is precluded by more systematic review methods.

## Social Policies

Individuals with lower incomes or precarious employment often face competing financial, relational, housing, transportation and health challenges that limit their access to healthy foods [10], with single parents, women, young people and migrants among those at heightened risk [16]. When food budgets are constrained, research shows that individuals can be swayed to purchase the cheapest and most discounted food and beverage options, which are commonly unhealthy [17]. Social policies, such as cash transfers (conditional and unconditional), income and employment support programs, housing and utilities assistance, address upstream structural determinants of healthy eating and food security, and can increase household resources to access healthy food [18, 19]. When designed with health and equity considerations in mind, these policies have been found to reduce food insecurity, enhance dietary quality and obesity prevention efforts, including in low- and middle-income countries [18].

The largest real-world conditional cash transfer program— Brazil’s Bolsa Familia Program— has operated since 2003, providing money to families living in poverty whose children attend school and health appointments. It is a national initiative of the Ministry of Social Development and Assistance with municipal implementation. Evaluation using 2017-18 data indicated that 87% of families used the program to purchase food, with most improving the variety of foods they consumed, especially in households experiencing food insecurity [20]. In an earlier study (2008-09), families enrolled in the program were more likely to purchase minimally processed foods and ingredients such as vegetables and meats, and did not differentially purchase ultra-processed foods and beverages, compared to families not in the program [21]. In a sample of pregnant women, participation in Bolsa Familia was associated with lower consumption of refined grains, salted meats and snacks, and a lower BMI compared to non-beneficiaries [22]. Furthermore, analysis of a cohort study found that Bolsa Familia beneficiaries who were followed for an average of 2.6 years, experienced small reductions in premature cardiovascular disease (Hazard Ratio (HR) = 0.96, 95% CI = 0.92-1.00) and all-cause mortality (HR = 0.96, 95% CI = 0.94–0.98), with the strongest associations among those in the lowest socioeconomic positions [23].

In Bangladesh, the Income Support Program for the Poorest (2014–2022) was designed to be gender sensitive by targeting mothers with the lowest incomes who had a child below 60 months of age [24]. Also implemented at the local level and conditional on attending child health and nutrition appointments, the program had high uptake and was modelled by the World Bank to have a positive net value of between $US174M—$US1.14B and return on investment of 13.9–20.2% [24]. Importantly, the program was modelled off a pilot that showed associations between the additional income and increased household dietary quality, although this outcome was not included in the long-term evaluation [24].

Although less studied, other forms of social support can also influence diet‑related health. In Japan, participation in the child allowance program was associated with children having half the odds of overweight compared with non‑beneficiaries [25]. Evidence from Canada’s Ontario Basic Income Pilot, one of few real-world universal income programs, likewise showed self‑reported improvements in nutrition among participants [26]. Overall, social policy interventions show some promising impacts on diet-related health outcomes, but the evidence remains limited to a small number of studies, partly due to short programme durations and challenges in capturing robust data. Continued government-funded evaluations, trials and longitudinal monitoring of diet, BMI and food security outcomes are needed to fully understand the contributions of these essential policies to improving public health nutrition.

## Education Policy

Schools are widely regarded as important settings for addressing overweight and food security because they can reach most children in many countries and provide opportunities for population-level nutrition strategies. Global guidance from the World Health Organization (WHO), United Nations Educational, Scientific and Cultural Organization (UNESCO), the United Nations Children’s Fund (UNICEF) and the Food and Agriculture Organization (FAO) recommends a range of school nutrition policies, including: provision of healthy school meals and safe drinking water; mandatory nutrition standards for foods and beverages provided or sold in schools; regulation of unhealthy foods, beverages and commercial marketing in and around schools; integration of nutrition education and health services within schools [27–29]. School nutrition policies are inherently multi-sectoral with education ministries leading development, implementation and monitoring; health authorities setting nutrition standards and monitoring student health; school leaders operationalising policies; and local governments regulating vendors and supporting local procurement. Although global evaluations remain limited, particularly in low- and middle-income countries, the WHO’s umbrella review of 117 systematic reviews found comprehensive, multi-component school nutrition policies were associated with reduced BMI and improved dietary outcomes, especially in low socioeconomic communities [30].

Japan’s Basic Law on Shokuiku (2005) [31] is a world-leading example of a national food and nutrition education policy framework led by the Ministry of Education, Culture, Sports, Science and Technology [32]. Integrated with the School Lunch Act (2008) [33], it mandates nutritionally compliant, freshly prepared school meals using local ingredients and uses lunchtime as a “living textbook” for sharing healthy eating responsibilities [34, 35]. Implementation involves the Ministry of Agriculture, Forestry and Fisheries for local procurement, whilst local governments resource school meals, local education boards oversee compliance at regional and school levels, and teachers, dietitians and families reinforce nutrition education [36]. The limited available evidence suggests participation in the school lunch program may be associated with a lower prevalence of overweight and obesity among boys (but not girls) or have no association overall [37, 38]. Despite this, Japan’s coordinated, multisectoral approach is considered a contributing factor for keeping childhood obesity rates low and fostering healthy diets into adulthood [39–42].

Indeed, school meal programs are gaining momentum around the world as a core initiative to improve the diets and food security priority groups of children. New Zealand’s Ka Ora, Ka Ako programme was implemented in 2020 to reduce food and educational inequities, providing free school lunches to the 25% of schools with the highest equity needs, reaching approximately 236,000–242,000 students daily [43]. Evaluations show students who previously experienced food insufficiency report feeling 20% fuller after lunch and that the meals contributed over one-third of daily nutrient requirements [44]. Additional data highlight reduced financial burden on families and reduced barriers to education, underscoring the program’s broader role as a targeted equity intervention [44]. Brazil’s National School Feeding Program (PNAE) has also aimed to increase food and nutrition security for students over several decades, with additional mandates for municipalities to procure at least 30% of foods from family farmers [45].

In other countries, including Chile and Mexico, school nutrition policies operate alongside broader healthy food environment regulations. Chile’s Food Labelling and Advertising Law combines front‑of‑pack warning labels with bans on selling or marketing unhealthy foods in and around schools. Although individual aspects of the policy cannot be isolated for research, evaluation has shown overall declines in purchased energy, sugar, saturated fat and sodium [46]. Mexico also prohibits the sale and marketing of foods carrying warning labels within schools, with some jurisdictions extending restrictions to nearby vendors and advertising, with early qualitative evidence suggesting reduced exposure to unhealthy food marketing [47]. Nonetheless, persistent implementation challenges include limited monitoring capacity, continued vendor sales of unhealthy foods and beverages, and inconsistent enforcement outside schools [47]. Together, these insights demonstrate that while education-sector food policies are logical solutions to improve food security and prevent obesity, their long-term success depends on stronger implementation systems. An expanded evidence base that more clearly demonstrates the long-term impacts on children’s health and cross-sector benefits is also needed.

## Economic Policies

Food and beverage prices and their affordability are structural determinants of population purchasing and consumption behaviours. Taxes and other economic measures can level out the relative price and affordability of foods and beverages to encourage healthier purchasing, especially among people in lower socioeconomic positions [48], whilst also generating revenue for public health programs [48]. Although often advocated for by health officials, economic policies are implemented and led by national treasury, finance or revenue departments, whose priorities can often drive policy decisions.

SSB taxes constitute some of the most widely researched public policies to improve population diets [49, 50]. Evidence synthesising the impacts of real-world SSB taxes from 86 studies found that they increased SSB prices (82% passed through to consumers) and reduced sales by 15% (95% CI: 9%, 20%) [49]. Tiered SSB taxes can also influence reformulation, as shown in the UK, with the total amount of sugar in sugary drinks reducing by 35% between 2015 and 2019 [51]. A systematic review of 14 economic evaluations consistently found that SSB taxes generate healthcare cost savings and have low implementation costs of 2–7% [50]. SSB taxes also likely improve health outcomes most for people in lower socioeconomic positions [50]. Although lower compared to higher socioeconomic groups may pay a higher proportion of income towards tax, higher income groups tend to spend more in absolute terms and therefore contribute more towards the tax revenue [50, 52]. Whilst some governments express concerns over adopting taxes during cost-of-living crises, particularly due to perceived increases in grocery expenses, there is no evidence that SSB taxes adversely impact food security. Overall, SSB taxes can deliver multiple benefits for governments—equitable reductions in SSB purchasing, healthier product reformulation and public revenue generation—often using existing tax structures [53]. In some regions such as Sub-Saharan Africa, SSB taxes have been estimated to generate more revenue than alcohol taxes [54].

SSB taxes have been implemented at the local level in the USA for more than a decade. A 2023 meta-analysis of 26 experimental studies found SSB taxes reduced SSB consumption (typically measured as changes in purchasing) by 27% on average [55]. Revenue analyses from seven jurisdictional SSB taxes in the USA indicated that these taxes generated a mean annual revenue of approximately US $134 million, with 85% allocated to supporting communities experiencing health inequities, including non-white communities [56]. Tax revenue was mostly invested into community development and early childhood (67%), followed by health promoting activities (28%) such as healthy food subsidies, whilst administration only accounted for 5% of the revenue [56]. Seattle, Boulder and Philadelphia showed high consistency in spending revenue with original promises, while in Berkeley, funds—though not earmarked—were allocated by an expert panel with strong community input and multisector coordination [56].

In an older example of economic policy to enable healthy diets for all, Australia implemented a Goods and Services Tax (GST) exemption for basic goods, including fruits, vegetables, dairy, bread, eggs, water and cooking ingredients [57] in 2000 (58). The exemption was designed by the Department of the Treasury, with support from the Office of Parliamentary Counsel, and implemented by the Australian Tax Office, with a view to create a more equitable tax system that ensures people on lower incomes can afford basic foods [58]. Economic modelling has indicated that removing this exemption would reduce fruit and vegetable demand, leading to an estimated 90,000 additional cases of major chronic diseases and AU $1 billion in lifetime healthcare costs [59]. Many countries also apply subsidies and price controls to protect against fluctuations and support food security [60]. However, a review of price controls in ten countries found that they can often include foods and beverages of low nutritional quality and negatively impact small-scale producers, showing the importance of ongoing efforts to strengthen policy coherence across health and economic departments [60].

## Trade and Agricultural Policies

Trade and agricultural support policies, often coordinated and implemented by departments of trade, agriculture and finance, include both trade and market interventions (e.g. border measures) as well as (dis)incentives for crop production (e.g. supports for farmers, input/output subsidies) [62]. Rather than directly shaping consumer prices, these policies influence what foods are produced, how they move through supply chains and ultimately their availability, price and diversity [61]. UN agencies have raised concerns that current policies often favour commodity crops and processed food ingredients, such as high-fructose corn syrup or maize-derived sweeteners [61, 62], while providing comparatively limited support for diversified production systems that could increase the availability and affordability of fruits and vegetables across populations [61, 62].

Looking at trade policies first, evidence has established associations between a country’s level of trade openness (i.e. the degree to which a country allows goods and services to move across its borders with minimal restrictions) and national obesity prevalence. A longitudinal analysis between 1975 and 2016 found that a 10% rise in trade openness was associated with a 0.8% (95%CI: 0.7–0.9%) increase in obesity across 175 countries, particularly in lower middle-income economies [63]. Entry into free trade agreements with the USA has likewise been associated with increases of 4.4% points in adult obesity prevalence [64]. It is thought that these associations are likely driven by increased availability and lower prices of highly processed foods, with the strongest effects in countries with lower incomes [62] and in small island states dependent on food imports [65]. To address these challenges, the UN has long recommended that policy coherence be strengthened between trade and nutrition; ensuring trade negotiations reflect nutrition, food security and economic objectives. This has been exemplified in the Pacific Islands for more than two decades, where Fiji, Samoa and Tonga used existing trade structures to restrict imports of fatty meats to promote population health [65].

In more recent times, the UN has called for national agricultural supports to be repurposed towards investments in healthier, more sustainable and equitable food systems [61]. The UN estimates that agricultural producers receive US$540 billion in government supports each year, 70% of which promotes commodity production, particularly sugar, rice, dairy, and meat, and 87% potentially distorting prices or harming human and planetary health [61]. The repurposing agenda focuses on the need to phase out these dominant supports and invest in diversifying production of fruits, vegetables, and legumes [66], which could increase consumption by up to 10% in Organisation for Economic Co-operation and Development (OECD) countries and deliver notable health and economic benefits [67]. Modelling further suggests that direct income payments (as a form of subsidisation) to farmers or consumers can help improve food security and poverty in low-income countries over the short and long-term [68].

Several countries are leading reforms to agricultural support policies, though evidence of their impacts on diets and obesity prevention remains limited. As previously alluded to, Brazil’s school meals program is supported by the Food Acquisition Program, which has been operating since 2003, originally implemented by the Ministry of Social Development, Assistance, Family and Combat against Hunger in coordination with the Ministry of Agriculture, Livestock and Food Supply. As a public procurement policy, local governments invest in buying food from farmers and small-scale producers for public feeding programs. Evidence has found a 13% increase in participating farmers’ agricultural income, especially for lower-income and small-scale farmers [69]. In recent years, Senegal’s Ministry of Agriculture and Rural Equipment has focused on reforming input subsidies to support greater use of agroecological fertilisers and diversification of seed subsidies beyond groundnuts (which have historically been central to Senegal’s agricultural economy and support systems) towards rice, onions, and diverse fruits and vegetables such as tomatoes and sweet potatoes [70]. This is reinforced by national strategies to improve the food and nutrition security of the population through enhanced trade, climate-resilient agricultural practices, and improved rural incomes and employment opportunities. Whilst improved input subsidies were shown to increase rice yield efficiency in Senegal [71], further evaluation is needed to assess the impacts of the wider diversification reforms and to determine how agricultural policies can best ensure equitable access to nutritious foods across contexts.

## Legal Policies

Legal and regulatory systems underpin the development, implementation and enforcement of many of the policies mentioned above such as tax reforms and trade measures. This section focuses on how legal and regulatory systems themselves can be strengthened or leveraged to reform food systems practices, including protecting consumers’ rights to safe and nutritious foods and beverages, regulating food industry practices, and enforcing compliance when industry actors breach regulations. Such efforts are necessary given that mandatory regulatory systems tend to support better public health outcomes than food industry self-regulation [72].

In Australia, the Departments of the Prime Minister and Cabinet and Treasury have introduced a series of legislative changes since 2020 to address food and cost-of-living inflation, which disproportionately impacted healthy compared to unhealthy foods and lower income households [73]. Legislative reforms included updating and mandating the Food and Grocery Code [74], passing laws to prohibit price gouging (with fines of up to AU$10 million for breaches after July 2026) and strengthening the Unit Pricing Code (to display unit prices and help consumers compare product prices) [74]. The Australian Competition and Consumer Competition (who enforce competition and consumer protection laws) also received additional funding to monitor food pricing practices and enforce these changes [74]. Meanwhile, in the USA, the Federal Trade Commission in 2022 reviewed a proposal for the largest supermarket merger in the country’s history between Kroger-Albertsons. The Federal Trade Commission alleged that the merger would reduce supermarket competition (which is considered unlawful) due to the existing high concentration, increase grocery prices and harm grocery workers – leading to a federal court ordering a temporary ban on the merger in 2024 and the supermarket companies no longer pursuing it [75]. Whilst these examples from the USA and Australia were implemented in response to industry competition and public cost-of-living concerns, they are likely to be win-wins for equitable obesity prevention and food security by ensuring food industries do not excessively price staple foods such as fruits and vegetables. Further investigations on the impact of these policies on diet intakes, obesity prevalence and food security will be needed in due course.

Beyond legislation and regulation, litigation is the process of resolving disputes through the court system and can also shape food policy by testing whether public-health regulations withstand legal challenge. In the UK, Kellogg’s brought a judicial review against obesity-prevention regulations restricting promotions of foods high in fat, salt or sugar [76]. The court upheld the government’s authority to introduce the measures, thereby reinforcing regulatory powers to protect public health. In the USA, lawsuits filed since 2024, including by the State of California, have alleged that ultra-processed food manufacturers contribute to diet-related harms through the addictive nature of their products, which are directly linked to obesity and related NCDs, drawing parallels with historic tobacco litigation [77, 78]. Although the case’s outcome is pending, it shows the need for strong evidence on food industry-related harms and robust legal and regulatory systems capable of counterbalancing industry power to favour community interests.

## Conclusions

This article contributes an overview of how government sectors beyond health can not only contribute to, but often lead, structural policy changes that are needed to concurrently advance social equity, food security, obesity prevention, sustainability and economic outcomes. Rather than providing an exhaustive list of actions, the review showcases real-world cross-sector policies and their impacts, moving beyond theory to illustrate practical opportunities for more mutually beneficial policy approaches. Table 1 summarises key recommendations for policymakers, researchers and practitioners in designing, implementing or evaluating these types of cross-sector policy actions.

**Table 1:** Summary of recommendations to advance equitable obesity prevention and food security policy beyond the health sector

Policy domain	Recommendations
Social policy	• Adopt or strengthen cash transfer programs for priority communities (e.g. women and children, young people, migrants and refugees) and periodically adjust them in response to current cost-of-living pressures and emerging crises. • Establish and resource government-led monitoring of the diet, food security, weight and broader population impacts of cash transfer programs over time to understand their effectiveness across contexts – thereby informing continuous policy improvement.
Education policy	• Institutionalise publicly funded, universal school feeding programmes, guided by nutrition standards. • Strengthen regulation of unhealthy food sales and marketing within and around schools. • Integrate nutrition education and health services in schools and communities. • Implement routine monitoring of school nutrition environments to track progress and assess impacts on dietary outcomes, food security and weight status–using data to guide education-sector obesity prevention and food security efforts across contexts.
Economic policy	• Implement (or advocate for the implementation of) SSB taxes using existing tax structures and allocate the revenue generated toward policies and programs that support the wellbeing of priority communities. • Explore options to maintain or expand fiscal policies such as GST exemptions and subsidies that improve affordability of minimally processed nutrient-rich foods.
Trade and agricultural policy	• Increase policy coherence between trade, agriculture and nutrition policy to align and ensure objectives deliver co-benefits. • Repurpose current government agricultural subsidies towards the production of healthy and sustainable foods and develop case studies to illustrate implementation pathways and co-benefits for other governments. • Enhance support for local production of minimally processed, nutrient-rich foods to increase healthy food availability and reduce reliance on imported highly processed foods.
Legal policy	• Strengthen and utilise legal and regulatory systems to safeguard the public from potentially harmful food industry practices. • Evaluate how litigation influences food industry regulation over time and the extent to which it advances community interests and public health objectives.

Achieving coordinated, cross-departmental policy to reduce overweight and obesity and improve food security will depend on better strategic alignment, political commitment and internal structures that connect stakeholders, enable effective communication, and enhance capacity [15]. Evidence further suggests that there is no one approach that will be effective across contexts, with some countries developing multi-sector departments/ministries, councils or other coordination entities to improve policy coherence [79]. From an equity perspective, participatory mechanisms that include non-government actors and community experience are also key to ensuring that policies across sectors, whether targeted or universal, ultimately reflect lived realities of food-related inequities and their structural determinants [80].

However, progressing these cross-sector policies is often impeded by siloed policymaking, nutrition and health advocates facing challenges with influencing actors outside their sectors, inadequate political leadership, and cross-sector coordination frequently weakening after policies are adopted [15, 79, 81]. In a recent study mapping multi-sectoral food systems actions across 190 countries, the unequal power and influence of different government departments were discussed, with departments of finance and trade often having stronger authority in policy decision-making than health or environment [79]. To date, this imbalance in resources and power has been largely to the detriment of obesity prevention, nutrition and food security policies, and consequently longer-term economic interests. As such, addressing nutrition inequities through a cross-sector agenda will require effective accountability mechanisms to ensure greater cooperation across sectors with different food system interests [81, 82]. Now is the time to rebalance policy by learning from global successes and strengthening cross‑departmental collaboration under a shared agenda to redesign policies that address present and future food and health challenges across populations.

## Data Availability

No datasets were generated or analysed during the current study.

## References

[1] Institute for Health Metrics and Evaluation (IHME). GBD 2021 Cause and Risk Summary: Dietary risks—Level 2 risk. Accessed 7th Nov 2024. Seattle, USA: IHME, University of Washington. 2024; 2024.

[2] Australian Institute of Health and Welfare. Overweight and obesity. Canberra 2024. Available from: https://www.aihw.gov.au/reports/overweight-obesity/overweight-and-obesity/contents/summary.

[3] Nisbett N, Harris J, Backholer K, Baker P, Jernigan VBB, Friel S. Holding no-one back: The Nutrition Equity Framework in theory and practice. Global Food Secur. 2022;32:100605. 10.1016/j.gfs.2021.100605 PMC9983632 36873709

[4] Needham C, Orellana L, Allender S, Sacks G, Blake M, Strugnell C. Food retail environments in Greater Melbourne 2008–2016: longitudinal analysis of intra-city variation in density and healthiness of food outlets. Int J Environ Res Public Health. 2020;17(4):1321. 10.3390/ijerph17041321 PMC7068484 32092853

[5] Needham C, Sacks G, Orellana L, Robinson E, Allender S, Strugnell C. A systematic review of the Australian food retail environment: characteristics, variation by geographic area, socioeconomic position and associations with diet and obesity. Obes Rev. 2020;21:e12941 10.1111/obr.12941 31802612

[6] Needham C, Strugnell C, Allender S, Orellana L. Beyond food swamps and food deserts: exploring urban Australian food retail environment typologies. Public Health Nutr. 2022;25(5):1–13. 35022093 10.1017/S136898002200009X PMC9991784

[7] Needham C, Strugnell C, Orellana L, Allender S, Sacks G, Blake MR, et al. Using spatial analysis to examine inequalities and temporal trends in food retail accessibility. Public Health Nutr. 2024;27(1):e222. 39445498 10.1017/S1368980024001344 PMC11604324

[8] Carrillo-Alvarez E, Rifà-Ros R, Salinas-Roca B, Costa-Tutusaus L, Lamas M, Rodriguez-Monforte M. Diet-Related Health Inequalities in High-Income Countries: A Scoping Review of Observational Studies. Adv Nutr. 2025;16(6):100439. 40334986 10.1016/j.advnut.2025.100439 PMC12149430

[9] Eskandari F, Lake AA, Rose K, Butler M, O’Malley C. A mixed-method systematic review and meta-analysis of the influences of food environments and food insecurity on obesity in high-income countries. Food Sci Nutr. 2022;10(11):3689–723. 36348796 10.1002/fsn3.2969 PMC9632201

[10] Zorbas C, Browne J, Chung A, Peeters A, Booth S, Pollard C, et al. Shifting the social determinants of food insecurity during the COVID-19 pandemic: the Australian experience. Food Secur. 2023;15(1):151–70. 36160693 10.1007/s12571-022-01318-4 PMC9483265

[11] Zorbas C, Browne J, Chung A, Baker P, Palermo C, Reeve E et al. National nutrition policy in high-income countries: is health equity on the agenda? Nutr Rev. 2021;79(10):1100–1113. 10.1093/nutrit/nuaa120 33230539

[12] World Health Organization. Reducing the burden of noncommunicable diseases through strengthening prevention and control of diabetes. WHA74.4. Geneva: World Health Assembly. 2021. Available from: https://apps.who.int/gb/ebwha/pdf_files/WHA74/A74_R4-en.pdf.

[13] Pescud M, Friel S, Lee A, Sacks G, Meertens E, Carter R, et al. Extending the paradigm: a policy framework for healthy and equitable eating (HE2). Public Health Nutr. 2018;21(18):3477–81. 30124178 10.1017/S1368980018002082 PMC10260926

[14] Kumanyika SK. A Framework for Increasing Equity Impact in Obesity Prevention. Am J Public Health. 2019;109(10):1350. 31415203 10.2105/AJPH.2019.305221 PMC6727309

[15] Parsons K. How connected is national food policy in England? Mapping cross-government work on food system issues. Centre for Food Policy, City, University of London. 2021. Available from: https://foodresearch.org.uk/publications/how-connected-is-national-food-policy-in-england-mapping-cross-government-work-on-food-system-issues/.

[16] Bowden M. Understanding food insecurity in Australia. CFCA PAPER NO. 55. Australian Government. Australian Institute of Family Studies. Child Family Community Australia. 2020. Available from: https://aifs.gov.au/sites/default/files/publication-documents/2009_cfca_understanding_food_insecurity_in_australia_0.pdf.

[17] Bennett R, Zorbas C, Huse O, Peeters A, Cameron A, Sacks G, et al. Prevalence of healthy and unhealthy food and beverage price promotions and their influence on consumer purchasing behaviour– a systematic review of the literature. Obes Rev. 2019;21(1):e12948. 31633289 10.1111/obr.12948

[18] Durao S, Visser ME, Ramokolo V, Oliveira JM, Schmidt BM, Balakrishna Y, et al. Community-level interventions for improving access to food in low- and middle-income countries. Cochrane Database Syst Rev. 2020;7(7):Cd011504. 32722849 10.1002/14651858.CD011504.pub2 PMC7390433

[19] McKay FH, Bennett R, Dunn M. How, why and for whom does a basic income contribute to health and wellbeing: a systematic review. Health Promot Int. 2023;38(5):daad119. 10.1093/heapro/daad119 37804514

[20] Figueroa-Pedraza D. Repercusiones del Programa Bolsa Família en la seguridad alimentaria y nutricional de familias en el estado de Paraíba, Brasil, 2017–2018. Revista Facultad Nac de Salud Pública. 2022;40(3).

[21] Martins AP, Monteiro CA. Impact of the Bolsa Família program on food availability of low-income Brazilian families: a quasi experimental study. BMC Public Health. 2016;16(1):827. 27538516 10.1186/s12889-016-3486-y PMC4991072

[22] Santana JDM, Pereira M, Lisboa CS, Santos DB, Oliveira AM. Influence of conditional cash transfer program on prenatal care and nutrition during pregnancy: NISAMI cohort study. Sao Paulo Med J. 2022;140(4):595–603. 35946676 10.1590/1516-3180.2021.0449.R1.23112021 PMC9491472

[23] Pescarini JM, Campbell D, Amorim LD, Falcão IR, Ferreira AJF, Allik M, et al. Impact of Brazil’s Bolsa Família Programme on cardiovascular and all-cause mortality: a natural experiment study using the 100 Million Brazilian Cohort. Int J Epidemiol. 2022;51(6):1847–61. 36172959 10.1093/ije/dyac188 PMC9749722

[24] IEG Review Team. Bangladesh - Income Support Program for the Poorest (English). Washington, D.C.: World Bank Group. http://documents.worldbank.org/curated/en/099061323090589446.

[25] Koyama Y, Fujiwara T, Isumi A, Doi S. Is Japan’s child allowance effective for the well-being of children? A statistical evaluation using data from K-CHILD study. BMC Public Health. 2020;20(1):1503. 33023534 10.1186/s12889-020-09367-0 PMC7542372

[26] Hamilton L, Mulvale JP. Human Again: The (Unrealized) Promise of Basic Income in Ontario. J Poverty. 2019;23(7):576–99.

[27] World Health Organization (WHO) and the United Nations Educational and Scientific and Cultural Organization (UNESCO). Making every school a health-promoting school: implementation guidance. Geneva; 2021.

[28] UNICEF, Nutrition, for Every Child.: UNICEF Nutrition Strategy 2020–2030. New York. 2020. https://www.unicef.org/reports/nutrition-strategy-2020-2030

[29] Food and Agriculture Organization of the United Nations. School Food and Nutrition. In: FAO, editor; 2025.

[30] Nutrition action in schools: a review of evidence related to the Nutrition-Friendly Schools Initiative. Geneva: World Health Organization; 2020. https://iris.who.int/server/api/core/bitstreams/33daf907-ef6c-4674-97a7-5f4cc67db97a/content.

[31] Miyoshi M, Tsuboyama-Kasaoka N, Nishi N. School-based Shokuiku program in Japan: application to nutrition education in Asian countries. Asia Pac J Clin Nutr. 2012;21(1):159–62. 22374574

[32] Oudat Q, Messiah SE, Ghoneum AD. A Multi-Level Approach to Childhood Obesity Prevention and Management: Lessons from Japan and the United States. Nutrients. 2025;17(5):838. 40077708 10.3390/nu17050838 PMC11902064

[33] Government of Japan. (2008). School Lunch Act (Act No. 160 of 1954, amended in 2008). Retrieved from https://www.fao.org/faolex/results/details/en/c/LEX-FAOC238793.

[34] Global Child Nutrition Foundation. How Japan leverages school meals as a “living textbook” for lifelong healthy eating. (Internet) https://gcnf.org/how-japan-leverages-school-meals-as-a-living-textbook-for-lifelong-healthy-eating/.

[35] Tanaka N, Miyoshi M. School lunch program for health promotion among children in Japan. Asia Pac J Clin Nutr. 2012;21(1):155–8. 22374573

[36] Japan International Cooperation Agency. Shokuiku: the key to nurturing healthier choices. Pan International Cooperation Agency, Human Development Department; 2024 (internet) https://www.jica.go.jp/english/activities/issues/nutrition/__icsFiles/afieldfile/2025/03/22/3_JICA_EN_Technical_Brief_Shokuiku.pdf?utm_source=chatgpt.com.

[37] Miyawaki A, Lee JS, Kobayashi Y. Impact of the school lunch program on overweight and obesity among junior high school students: a nationwide study in Japan. J Public Health (Oxf). 2019;41(2):362–70. 29873776 10.1093/pubmed/fdy095 PMC6636685

[38] Iwano S, Tanaka K, Takaoka A, Machida D, Tomata Y. School lunch and body size in Japanese junior high school students: the Japanese National Health and Nutrition Survey. Nutrients. 2025;17(5):895. 40077765 10.3390/nu17050895 PMC11901768

[39] Japan National Health and Nutrition 2019 Survey. Obesity Prevalence. 2019. Available online: https://www.e-stat.go.jp/stat-search/files?page=1&layout=datalist&toukei=00450171&tstat=000001041744&cycle=7&tclass1=000001148507&tclass2val=0.

[40] Kuwahara M, Eum W. Effects of Childhood Nutrition Education from School and Family on Eating Habits of Japanese Adults. Nutrients. 2022;14(12):2517. 35745246 10.3390/nu14122517 PMC9230025

[41] Kibayashi E, Nakade M. Associations between Shokuiku during School Years, Well-Balanced Diets, and Eating and Lifestyle Behaviours in Japanese Females Enrolled in a University Registered Dietitian Course. Nutrients. 2024;16(4):484. 38398808 10.3390/nu16040484 PMC10892923

[42] Kaneda M, Yamamoto S. The Japanese School Lunch and Its Contribution to Health. Nutr Today. 2015;50(6):268–72.

[43] Ministry of Education. Healthy School Lunches Programme – Ka Ora, Ka Ako. https://www.education.govt.nz/our-work/overall-strategies-and-policies/wellbeing-in-education/free-and-healthy-school-lunches.

[44] Mejía Toro C, King J, Mackay S, et al. Free, healthy school lunches in New Zealand: A Value for Investment analysis. BMC Public Health. 2025;25:3546. 10.1186/s12889-025-24529-8. 41121057 10.1186/s12889-025-24529-8 PMC12539167

[45] Martinez P, de Lourdes Saturnino Gomes M, Marini FS. Public policies strengthen the relationship between family farming and food security in Brazilian schools - A case study of Paraíba state. Heliyon. 2023;9(10):e20482. 37810807 10.1016/j.heliyon.2023.e20482 PMC10556778

[46] Taillie LS, Bercholz M, Popkin B, Reyes M, Colchero MA, Corvalán C. Changes in food purchases after the Chilean policies on food labelling, marketing, and sales in schools: a before and after study. Lancet Planet Health. 2021;5(8):e526–33. 34390670 10.1016/S2542-5196(21)00172-8 PMC8364623

[47] Hugues Y, Díaz-Zavala RG, Quizán-Plata T, Corvalán C, Haby MM. Poor compliance with school food environment guidelines in elementary schools in Northwest Mexico: A cross-sectional study. PLoS ONE. 2021;16(11):e0259720. 34762702 10.1371/journal.pone.0259720 PMC8584694

[48] World Health Organization. WHO manual on sugar–sweetened beverage taxation policies to promote healthy diets. Geneva: World Health Organization; 2022. Available from: https://www.who.int/publications/i/item/9789240056299.

[49] Andreyeva T, Marple K, Marinello S, Moore TE, Powell LM. Outcomes Following Taxation of Sugar-Sweetened Beverages: A Systematic Review and Meta-analysis. JAMA Netw Open. 2022;5(6):e2215276–e. 35648398 10.1001/jamanetworkopen.2022.15276 PMC9161017

[50] Thiboonboon K, Lourenco RDA, Cronin P, Khoo T, Goodall S. Economic evaluations of obesity-targeted Sugar-Sweetened Beverage (SSB) taxes–a review to identify methodological issues. Health Policy (Amsterdam, Netherlands). 2024;144:105076. 10.1016/j.healthpol.2024.105076 38692186

[51] Sasse T, Metcalfe S. Sugar tax. Institute for Government; 2022 Nov 14 [updated 2024 Jul 10]. Available from: https://www.instituteforgovernment.org.uk/explainer/sugar-tax.

[52] Jones-Smith JC, Knox MA, Coe NB, Walkinshaw LP, Schoof J, Hamilton D, et al. Sweetened beverage taxes: Economic benefits and costs according to household income. Food Policy. 2022;110:102277. 38031563 10.1016/j.foodpol.2022.102277 PMC10686549

[53] Forberger S, Reisch L, Meshkovska B, Lobczowska K, Scheller DA, Wendt J, et al. Sugar-sweetened beverage tax implementation processes: results of a scoping review. Health Res Policy Syst. 2022;20(1):33. 35331245 10.1186/s12961-022-00832-3 PMC8944035

[54] Summan A, Laxminarayan R. Global effects of increased taxation of tobacco, alcohol, and sugar-sweetened beverages on tax receipts: a modelling analysis. Prepared for the Task Force on Fiscal Policy for Health; 2024. Available from: https://assets.bbhub.io/dotorg/sites/64/2024/09/Health-Taxes-Modelling-Paper.pdf. 10.1136/bmjgh-2024-017571 PMC12716585 41419250

[55] Shen J, Wang J, Yang F, An R. Impact of soda tax on beverage price, sale, purchase, and consumption in the US: a systematic review and meta-analysis of natural experiments. Front Public Health. 2023;11:1126569. 37808982 10.3389/fpubh.2023.1126569 PMC10556476

[56] Krieger J, Magee K, Hennings T, Schoof J, Madsen KA. How sugar-sweetened beverage tax revenues are being used in the United States. Prev Med Rep. 2021;23:101388. 34040929 10.1016/j.pmedr.2021.101388 PMC8141925

[57] Australian Taxation Office (ATO). GST and food – GST-free food. Retrieved from https://www.ato.gov.au (Updated 2025, July 25).

[58] Kenny P. The GST food exemption. J Aust Tax. 2000;3(6):424–39.

[59] Veerman JL, Cobiac LJ. Removing the GST exemption for fresh fruits and vegetables could cost lives. Med J Aust. 2013;199(8):534–5. 24138373 10.5694/mja13.11064

[60] Sträuli B, Thow AM, Reeve E. Policy coherence of price controls on food and noncommunicable disease prevention, WHO South-East Asia and Western Pacific regions. Bull World Health Organ. 2025;103(1):43–50. 39781002 10.2471/BLT.24.291812 PMC11704635

[61] FAO, UNDP and UNEP. A multi-billion-dollar opportunity – Repurposing agricultural support to transform food systems. Rome FAO. 2021. 10.4060/cb6562en.

[62] Food and Agriculture Organization of the United Nations (FAO). The state of agricultural commodity markets 2024: trade and nutrition – Policy coherence for healthy diets. Rome: FAO; 2024. Available from: 10.4060/cd2144en.

[63] An R, Guan C, Liu J, Chen N, Clarke C. Trade openness and the obesity epidemic: a cross-national study of 175 countries during 1975–2016. Ann Epidemiol. 2019;37:31–6. 31399309 10.1016/j.annepidem.2019.07.002

[64] Baggio M, Chong A. Free trade agreements and world obesity. South Econ J. 2020;87(1):30–49.

[65] Thow AM, Swinburn B, Colagiuri S, Diligolevu M, Quested C, Vivili P, et al. Trade and food policy: Case studies from three Pacific Island countries. Food Policy. 2010;35(6):556–64.

[66] Reeve E, Mason-D’Croz D, Thompson Thow AM. Health sector advocacy for repurposing agricultural investments affecting fruits, vegetables and legumes. Bull World Health Organ. 2025;103(5):328–36. 40342846 10.2471/BLT.24.292201 PMC12057240

[67] Springmann M, Freund F. Options for reforming agricultural subsidies from health, climate, and economic perspectives. Nat Commun. 2022;13(1):82. 35013212 10.1038/s41467-021-27645-2 PMC8748512

[68] Brooks J, Filipski MJ, Jonasson E, Taylor JE. The distributional implications of agricultural policies in developing countries - findings from the Development Policy Evaluation Model (DEVPEM). Agricultural policies for poverty reduction. 2012:89.

[69] Casagrande D, Emanuel L, Freitas C, Lima A, Nishimura F, Oliveira F. Public food procurement and production: Evidence of the food acquisition program in Brazil. Food Policy. 2024;126:102656.

[70] FAO/FAOLEX. Programme d’Accélération de la Cadence de l’Agriculture Sénégalaise (PRACAS). UNEP Law and Environment Assistance Platform; [cited 2026 Jan 20]. Available from: https://leap.unep.org/en/countries/sn/national-legislation/programme-dacceleration-de-la-cadence-de-lagriculture-senegalaise.

[71] Diallo MF, Garba AA. Effect of seed and fertilizer subsidies on the technical efficiency of rice farmers in Senegal. 50x2030 Initiative. IFAD. 2023;16:1–81.

[72] UNICEF. UNICEF technical note on effective regulatory approaches to protect, support and promote better diets and create healthy food environments for children. New York: UNICEF; 2021.

[73] Vargas C, Backholer K, Bennett R, Maganti R, Lewis M, Marshall J et al. Working towards affordable healthy diets: a review on innovations in food price monitoring, policy and research in Australia and beyond. Proc Nutr Soc. 2025:1–12. 10.1017/S0029665125101225 40910430

[74] Leigh A. Consultation on making supermarket price gouging illegal [Internet]. Canberra: Australian Treasury; 2025 Oct 20 [cited 2026 Jan 16]. Available from https://ministers.treasury.gov.au/ministers/andrew-leigh-2025/media-releases/consultation-making-supermarket-price-gouging-illegal.

[75] Statement of Chair Lina M. Khan in the Matter of The Kroger Company and Albertsons Companies, Inc., Commission File No. D9428. Federal Trade Commission; 2 Jan 2025. Available from: https://www.ftc.gov/system/files/ftc_gov/pdf/2025.01.02-statement-of-chair-lina-m.-khan-in-the-matter-of-the-kroger-company-and-albertsons-companies-inc.-final.pdf.

[76] Food Foundation. Kellogg’s loses legal challenge to government food rules [Internet]. London: Food Foundation. 2022 Jul 4 [cited 2026 Feb 20]. Available from: https://foodfoundation.org.uk/press-release/kelloggs-loses-legal-challenge-government-food-rules.

[77] King R. Ultra–processed foods lawsuit – January 2026 update [Internet]. King Law; 2026 Jan 7 [cited 2026 Jan 16]. Available from: https://www.robertkinglawfirm.com/mass-torts/ultra-processed-foods-lawsuit/.

[78] Lytle J. The food wars and the courts [Internet]. Petrie–Flom Center; 2025 Apr 4 [cited 2026 Jan 16]. Available from: https://petrieflom.law.harvard.edu/2025/04/04/the-food-wars-and-the-courts/.

[79] Patay D, Rippin H, Ares G, Reeve E, Hargous CV, Farrell P, et al. From ministries of food to national food system committees: a global mapping and typology of multisectoral food system governance institutions. Sustain Dev. 2025;34(1):1378–98.

[80] Zorbas C, Jeyapalan D, Nunez V, Backholer K. Community lived experience should be central to food systems policy. Nat Food. 2023;4:7–9. 37118565 10.1038/s43016-022-00676-8

[81] Patay D, Reeve E, Thow AM, Baker P, Farrell P. Whole-of-food system governance for transformative change. Nat Food. 2025;6(7):636–40. 40646351 10.1038/s43016-025-01196-x

[82] Reeve E, Bell C, Sacks G, Mounsey S, Waqa G, Peeters A, et al. Lessons for strengthening policymaking for obesity and diet-related noncommunicable disease prevention: A narrative synthesis of policy literature from the Western Pacific Region. Obes reviews: official J Int Association Study Obes. 2024;25(2):e13651. 10.1111/obr.13651 37905309

